# MOBILITY AND PHYSICAL ACTIVITY IN OLDER ADULTS: OBSERVATIONAL AND INTERVENTION RESEARCH

**DOI:** 10.1093/geroni/igz038.1605

**Published:** 2019-11-08

**Authors:** Patricia M Bamonti, Jonathan Bean

**Affiliations:** 1 VA Boston Healthcare System, Boston, Massachusetts, United States; 2 New England GRECC, VA Boston Healthcare System, Boston, Massachusetts, United States

## Abstract

Mobility disability is associated with considerable morbidity and mortality in late life. Physical activity [PA] is a modifiable behavior that can reduce mobility disability, as well as improve physical and mental health outcomes in older adults. However, only a minority of older adults meet the minimum PA requirements based on national guidelines. Research examining factors impacting PA and mobility in late life, as well as novel interventions to increase PA and improve mobility is essential to enhancing health and wellbeing. This symposium will provide an overview of observational and intervention research focused on understanding factors associated with PA and mobility, as well as intervention research designed to increase PA and improve mobility in older adults. First, Dr. Lien Quach will present research examining the impact of social engagement in reducing the risk of mobility decline among older adults with mild cognitive impairment. Second, Dr. Patricia Bamonti will examine psychological factors related to uptake and adherence of pulmonary rehabilitation in older Veterans. Third, Dr. Stephanie Robinson will explore engagement, feasibility, acceptability, and change in PA following a web-based intervention in middle-aged and older adult patients with COPD. Fourth, Dr. Elisa Ogawa will present research examining the feasibility and acceptability of an exergaming intervention compared to an exercise control condition among older adults at risk for falls. The symposium will conclude with discussion led by Jonathan Bean, MD, MPH, who will highlight implications of findings across studies and identify areas for future research.

